# A Framework for Supervision for Mindfulness-Based Teachers: a Space for Embodied Mutual Inquiry

**DOI:** 10.1007/s12671-014-0292-4

**Published:** 2014-03-23

**Authors:** Alison Evans, Rebecca Crane, Lucinda Cooper, Jody Mardula, Jenny Wilks, Christina Surawy, Maura Kenny, Willem Kuyken

**Affiliations:** 1 Exeter Mindfulness Network, School of Psychology, University of Exeter, Exeter, EX4 4QG UK; 2 Centre for Mindfulness Research and Practice, School of Psychology, Bangor University, Gwynedd, LL57 1UT UK; 3 Department of Psychiatry, Warneford Hospital, University of Oxford, Oxford, OX3 7JX UK; 4 Centre for the Treatment of Anxiety and Depression, Central Adelaide Local health Network, 30 Anderson St, Thebarton, South Australia 5031 Australia

**Keywords:** Mindfulness-based interventions, Mindfulness-based stress reduction, Mindfulness-based cognitive therapy, Supervision, Framework, Training, Good practice guidance

## Abstract

Over recent decades, there has been an exponential growth in mindfulness-based interventions (MBIs). To disseminate MBIs with fidelity, care needs to be taken with the training and supervision of MBI teachers. A wealth of literature exists describing the process and practice of supervision in a range of clinical approaches, but, as of yet, little consideration has been given to how this can best be applied to the supervision of MBI teachers. This paper articulates a framework for supervision of MBI teachers. It was informed by the following: the experience of eight experienced mindfulness-based supervisors, the literature and understandings from MBIs, and by the authors’ experience of training and supervision. It sets out the nature and distinctive features of mindfulness-based supervision (MBS), representing this complex, multilayered process through a series of circles that denote its essence, form, content and process. This paper aims to be a basis for further dialogue on MBS, providing a foundation to increase the availability of competent supervision so that MBIs can expand without compromising integrity and efficacy.

## Introduction

With the upsurge in interest in mindfulness-based interventions (MBIs) for a range of populations across many different settings, more people are undertaking training to teach mindfulness-based stress reduction (MBSR) and mindfulness-based cognitive therapy (MBCT). This growth has created a bottleneck because although there has been increased demand for MBIs, there are a limited number of MBI teachers and supervisors. A report of commissioned surveys of service users and GPs (Mental Health Foundation 2010) made key recommendations, including expanding mindfulness-based training and supervision opportunities. Within the UK, training in MBSR and/or MBCT is available at three university centres, within some National Health Service (NHS) contexts and through several independent training organisations. Guidance for good practice in teaching MBSR and MBCT have consistently pointed to the centrality of supervision at all levels of experience.

To date, there is not a professional mindfulness-accrediting body. However, training organisations are coming together to agree on good practice guidelines. The UK Network for Mindfulness-Based Teacher Training Organisations has representation from all the main MBSR, MBCT and Breathworks training organisations within the UK. A recent priority has been to set out guidelines to promote good practice for teachers and trainers of teachers (UK Network of Mindfulness-Based Teacher Training Organisations 2011). In the teacher guidelines, supervision appears in the section “on-going good practice requirements”, under the second element, “engagement in processes which continue to develop mindfulness-based teaching practice”. The supervision section reads:

Regular supervision with an experienced mindfulness-based teacher, including:

i. Opportunity to reflect on/inquire into personal process in relation to personal mindfulness practice and mindfulness-based teaching practice.
ii. Receiving periodic feedback on teaching through video recordings, supervisor sitting in on teaching sessions or co-teaching with reciprocal feedback.

Crane et al. (2010) also emphasised how supervision is an integral part of the learning process from basic teacher training to continuing professional development. In the National Institute for Clinical and Health Excellence depression guideline (NICE 2009), under effective delivery of interventions for depression, we see the recommendation that all practitioners should receive high quality supervision. In addition, the Centre for Mindfulness in Medicine, Healthcare, and Society at the University of Massachusetts Medical School considers it to be one of the standards of practice that those who train teachers to deliver MBSR receive MBSR supervision from a certified teacher trainer (Kabat-Zinn and Santorelli 2011).

## Developing a Framework

In this paper, we propose a framework and description of mindfulness-based supervision (MBS) with the intention to present a conceptual view of what occurs in MBS. Our ideas have been developed from and informed by the MBI training programmes we work within, our personal mindfulness practice and the lineage of mindfulness practice and understanding, and the perspective of eight experienced supervisors (from descriptions of their experiences of giving and receiving MBS).

There is a diversity of frameworks and models of supervision for different therapeutic interventions. Although these have influenced MBS, it is beyond the scope of this paper to present detailed reviews of these. In brief, the literature that has informed our conceptual understandings has included the following: literature on the pedagogy of MBSR and MBCT teaching on which the process of MBS is based, literature on supervision in therapeutic contexts (which provided a useful reference point for consideration of core structures, frameworks and elements of supervision), and the limited literature on mindfulness supervision within counselling (with underlying intentions of being in the present moment).

From the outset, dialogue with a group of experienced mindfulness-based supervisors seemed an important part of the process of developing a conceptual framework about MBS. As part of a dissertation, the first author set up telephone interviews to begin to generate dialogue about the nature of MBS. To enable the process to be illustrative of UK MBS practice, five UK supervisors were chosen. Furthermore, to enable the process to be informed by practice in the international context, three international supervisors (Australia, Netherlands and USA) were chosen. All supervisors worked in organisations responsible for training and supervising MBSR/MBCT teachers. They had long-standing personal mindfulness practices and had been teaching MBSR/MBCT for between 8 and 18 years. These supervisors are referred to as interviewees or by a pseudonym when quoted. The first author works within a university setting with experience of supervising in the context of postgraduate training in mindfulness-based cognitive therapies and approaches, a National Institute for Health Research-funded trial, an NHS-funded clinic delivering MBCT/MBSR and with a range of independent MBCT/MBSR teachers as well as receiving MBS from a number of supervisors over the last 9 years.

The interviews explored the intentions, functions, content, structure and processes of MBS. They were recorded and transcribed, firstly, for further reference and secondly, to provide anonymised quotes to illustrate themes. The first author drew together a framework which was informed by the information from these interviews, themes generic to all supervision and her personal experience of MBS. This initial framework was adapted and refined by further exploration and dialogue with the coauthors, who also all work within organisations training in MBIs (several of them also supervise within wider contexts, e.g. NHS-funded clinical work, research trials and with independent practitioners).

## A Framework for Supervision of MBSR/MBCT Teachers

MBS integrates supervisory processes employed in other contexts with central aspects of MBSR and MBCT pedagogy. Two important aspects that emerged from the interviews were mindfulness as a key aspect of MBS and also the particular inquiry approach found within MBIs. These two distinctive features formed the foundation for the framework, along with other elements identified.

**Fig. 1:**
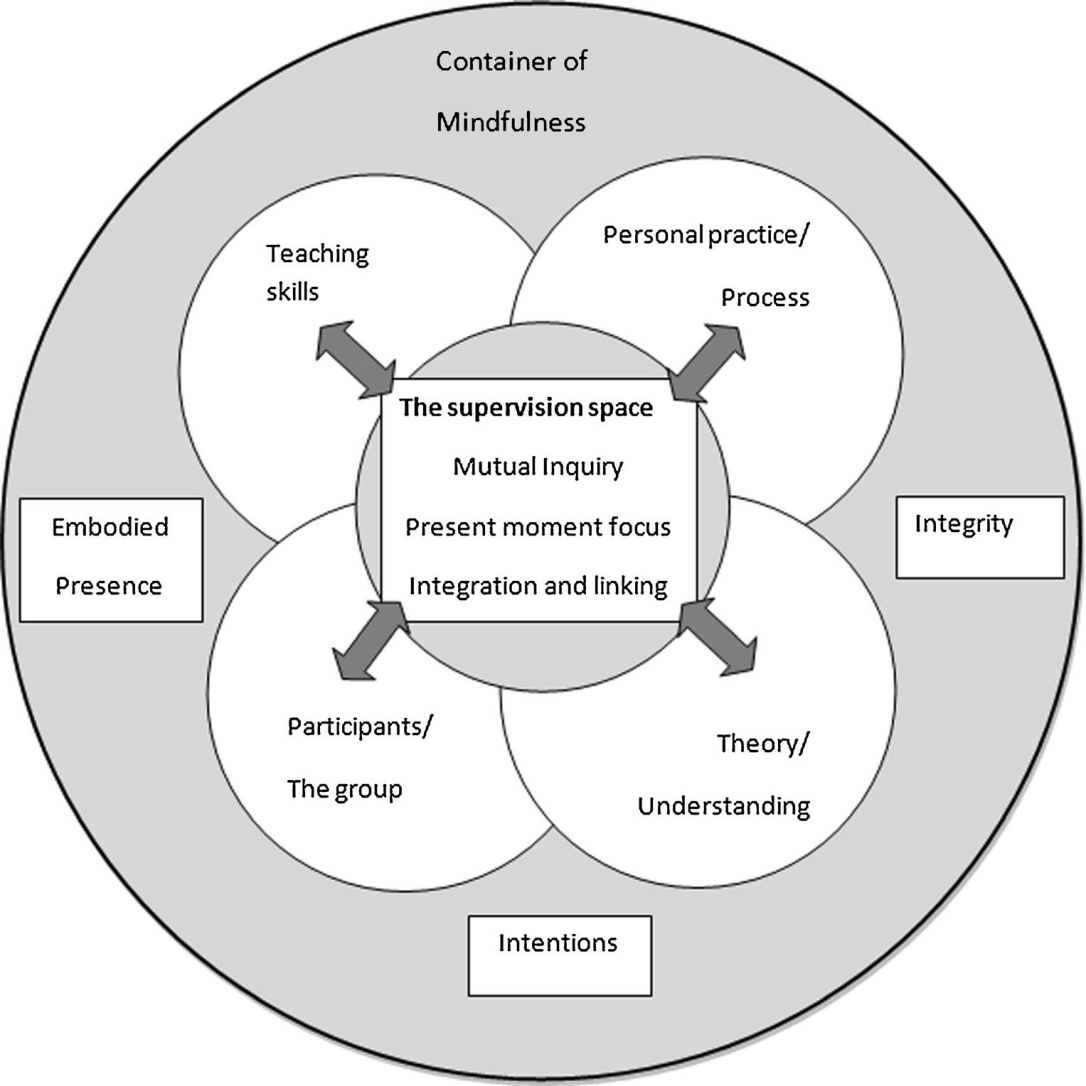
Framework for supervision of MBSR/MBCT teachers showing the distinctive features

This complex, multilayered process is represented in Fig. 1. A series of circles denotes the different layers of supervision. The outer circle is the essence of the process and practice of mindfulness that infuses the whole supervision process. The middle circles represent the form and content that is brought to supervision. The inner circle represents the process that happens within the supervision space to enable learning and integration.

## The Outer Circle—the Container of Mindfulness

A key aspect of MBS is the holding of everything that happens in supervision within a container of mindfulness. Crane et al. (2010) wrote, “The essential premise here is that the whole of the teaching (and training) process is mindfulness-based” (p.79). Likewise, the whole of supervision is mindfulness-based. By maintaining a thread of mindfulness, the supervisor plays an important role supporting teachers to sustain the intention to practice what they teach. The particular characteristics of this circle are the following:

### Embodied Presence

The essence of MBS is an embodiment of the principles and process of mindfulness. These have been represented by Kabat-Zinn (1990) as the attitudinal qualities of the following: non-judging (awareness of experience as it is, seeing with kindness when we are adding interpretation and judgement and stepping back), patience (allowing time for experience to unfold at its own pace), beginner’s mind (keeping a freshness and aliveness to the present moment), trust (developing a trust in the validity of our own experience and intuition), non-striving (moving into non-doing, letting things be as they are), acceptance (being with reality in a compassionate way) and letting go (coming back to immediacy of present experience, disentangling from unhelpful habits).

The way in which the teacher embodies the spirit and essence of the practices is a key ingredient of mindfulness-based classes (Crane et al. 2010). The teacher communicates this through their own sense of being. Supervision is also imbued with curiosity and a willingness to be fully present to whatever arises. The supervisor is likewise embodying the same spirit and so implicitly invites the supervisee to speak, listen, think, reflect, sit, feel, write and hold their body through a kindly and curious awareness of this present moment.

The movement of compassion towards suffering is an essential part of embodied mindfulness-based teaching. Research shows that increases in compassion and mindfulness are an important influence in changing the nature of the relationship between cognitive reactivity and outcome (Kuyken et al. 2010). Compassion has many different threads including kindness, empathy, generosity and acceptance as well as courage, tolerance and equanimity. The supervisor holds these qualities within the process of MBS, which in turn allows space for the supervisee to connect with compassion for themselves and their participants. This implicit embodiment of compassion mirrors how compassion is primarily conveyed within MBSR/MBCT. Therefore, it is important that teachers are sustained in their capacity to imbue their teaching with compassion. Effective supervision can challenge the supervisee to explore difficult places which, without the counter balance of compassion, may trigger reactive patterns of avoidance, fixing, judging and blaming. A compassionate atmosphere can guide the supervisee away from judgemental patterns towards a more creative and balanced space. Supervisees can begin to see “a thought as thought, an emotion as emotion, a habit as a habit and begin to take the ‘I’ out of the process” (Feldman and Kuyken 2011, p. 153).

Care must be taken to fully understand compassion and not misinterpret it as the need to be warm and supportive in a way that creates inappropriate comfort, as supervision can sometimes have a challenging edge to it as well. Several interviewees spoke of this important edge in the supervision process:

There is something that irritates me, that if you are a mindfulness teacher/supervisor you have to be lovely, warm, cuddly and compassionate. You know I can be and I am not. We do have a responsibility around holding, to be fierce and confront. We are holding the integrity here. (Emily)

A sense of common humanity is an important aspect of supervision by encouraging the teacher to include themselves in the teaching process:

An enormous amount of what I do is helping people to ground themselves and teach from their wobbles rather than trying to get rid of them. It is a practice that has to be developed over time. I don’t just say it, we do it. We do it a lot in the supervision session, we FOFBOC (Feet on floor, Bum on chair) (The Mindfulness in Schools Project 2013). And I encourage them to use that in their teaching, with any opportunity to teach from that wobble. Can I create an environment where we can play with that in supervision? And hopefully that can then be taken out into their work. (Diane)

### Integrity

A key guiding intention of supervision is upholding integrity through supporting and developing the supervisees’ teaching practice and how they relate to it. There are a number of elements of integrity that are addressed in supervision.

The integrity of the MBSR/MBCT programme itself is a central element of supervision. The Bangor, Exeter and Oxford mindfulness-based interventions: treatment assessment criteria (MBI: TAC) (Crane et al. 2012a, 2013; Crane et al. 2012b) is increasingly being used as a tool in supervision. The MBI: TAC describes six domains essential to the teaching of MBIs, namely coverage, pacing and organisation of session curriculum; relational skills; embodiment of mindfulness; guiding mindfulness practices; conveying course themes through interactive inquiry and didactic teaching; and the management of the group learning environment. As an assessment tool, it aims to assess integrity in three areas, namely the teacher’s competence (the extent to which the approach is carried out as originally intended), their level of adherence (the extent to which the teacher includes the key curriculum elements of an MBSR/MBCT course) and differentiation (the extent to which the teacher excludes elements that are not part of the MBSR/MBCT model and programme). In addition to being an assessment tool, it provides a structure and framework for teachers and supervisors to reflect on teaching with integrity.

In the pressured and outcome-orientated cultures in which many teachers are implementing mindfulness-based approaches, there may be drivers that push towards a dilution of integrity. This is described by some of the supervisors in the interviews and observed from our experience of training teachers, particularly within National Health Service (NHS) settings. It is important for supervisees to have space to explore how to respond to these potential dilutions with appropriate levels of firmness and flexibility.

Monitoring adherence to good practice is an important aspect of supervision because the supervisee may not have the experience to make clear judgements regarding integrity. The supervisor then plays an important role in helping them to be aware of the limits of their own competence. An interviewee spoke of this:

I supervised a new teacher who wanted to teach five MBSR courses all at once and instead of my saying, “Absolutely no, it’s impossible,” I said, “Well, can we maybe take it down to three?” I really explored with him what was going on within his body whilst we reflected on this. So it was more a sense of OK so maybe now you can see why this is a lot more complex and difficult than you thought. (Julia)

A function of supervision is to challenge practice that is poor, unethical for participants or unhealthy for teachers. At times, this requires a reflective exploration and, in some instances, a straight expression of a concern. This can be a difficult balance for supervisors who also have an intention to create a safe space for the supervisee to reflect openly. Importantly, there are some ethical processes that the supervisor cannot monitor so the contracting process needs to make sure these are taken care of (e.g. those working in health care settings will have clinical responsibility resting elsewhere.)

When considering integrity, it is also important to acknowledge and stay true to the underlying philosophy of mindfulness practice, which is based on the tradition of Buddhism, and to the MBSR and MBCT programmes that have developed from a deep understanding of this foundational base. Equally, it is important to be fully aware of the underlying theoretical and clinical roots of an approach such as MBCT. Mindfulness within secular settings, such as healthcare, is still a new field. As it grows, some interesting challenges arise around holding the essential essence of these programmes whilst also adapting for new populations and contexts. Supervision forms part of the process of shaping and monitoring future directions. As one of the interviewees stated, “In the back of my mind is always my role in preserving the integrity of the approach.” (Julia)

### Intentions

An important aspect of mindfulness practice is clarity of intention. This is mirrored in MBS, which aims to be clear regarding its intentions, to reconnect supervisees with their intentions for engaging in this work and, ultimately, provide a duty of care for the people in the class and the teacher. A range of intentions and functions of MBS were identified through the interviews.

We have drawn upon the influential ideas from other areas of supervision to categorise these intentions into three main functions. Kadushin (1976) described and defined these functions under the headings of Educational, Supportive and Managerial; Proctor (1988), also describes three areas, defining them as Formative, Restorative and Normative; and, based on these models, Hawkins and Shohet (2006) adapted this to their area and also defined three areas as Developmental, Resourcing and Qualitative. Using the three main functions in this way covers the breadth and depth of the intentions that were identified throughout all of the interviews. They have been combined together to form examples against the three main functions (Kadushin 1976; Hawkins and Shohet 2006; Proctor 1988) in Table 1. The functions and intentions may vary in prominence depending on the context that the supervisee is working within. For example, within an assessed training or research trial context, assessment and evaluation play a greater role, whilst the supportive function may be uppermost if the supervisee is teaching in an unsupportive context.

**Table 1:** Examples of intentions of supervision for MBSR/MBCT teachers presented under the three functions of supervision defined by Kadushin (1976), Proctor (1988) and Hawkins and Shohet (2006) (shown in this order in the first column)

Key functions	Examples of key intentions within each function as identified from interviews
*Educational* *Formative* *Developmental* Focus on learning and development	To stimulate curiosity and understanding of clients, the group, the self (teacher)—keeping alive a sense of inquiry to the whole process To develop and enhance skills in core competencies To deepen knowledge and understanding of concepts and theory and link with teaching To feedback on strengths and learning edges, which can then be incorporated into teaching To promote reflective practice for learning to take place allowing for choice points/different options
*Supportive* *Restorative* *Resourcing* Acknowledges the emotional and personal side of the work	To establish a good working relationship which is safe, supportive and nourishing—a place to unpack the impact of the work, overcome obstacles to learning, be creative and receive guidance in times of need To support being human, compassionate To support the development and deepening of ongoing personal mindfulness practice and its interface with teaching and everyday life To be in the present moment
*Managerial* *Normative* *Qualitative* The managerial and ethical issues	Assessment and evaluation To promote an ethical and safe practice to maintain standards and duty of care To be true to what is being taught—adhering to core curriculums and enabling mindfulness to be beneficial to people To engender a sense of responsibility for the supervisee and the people they are teaching To do no harm To challenge misunderstandings or poor practice

The developmental stage of the supervisee emerged as a feature that strongly shaped the supervision process. One of the key works describing a developmental approach to supervision comes from Stoltenberg and Delworth (1987), whose work holds that the process of supervision evolves as the supervisee develops their practice. As we use the MBI: TAC, we have been able to see how different levels of experience/training are linked to different competencies. Subsequently, it follows that different level of training/experience lead to differing needs and characteristics within MBS.

Table 2 shows a summary of the way we see stages of development aligned to the stages of competence in the MBI: TAC (Crane et al. 2012a, 2012b, 2013) and informed by a developmental approach to supervision described by Stoltenberg and Delworth (1987).

**Table 2:** Summary of the key characteristics of supervision at different developmental levels based on (Stoltenberg and Delworth 1987) and the MBI: TAC stages of competence (Crane et al. 2012a, 2012b, 2013)

Stage	Characteristics of supervisee	Characteristics of supervision sessions	Supervisor role
Beginner, advanced beginner and moving into competent	Inconsistencies around confidence May have feelings of insecurity or an over confidence	Clearly structured Practical skills-based Content- and curriculum-based Developing understandings of underlying intentions	Guidance Positive feedback Support Assessment
Competent	Inconsistencies around confidence may still be present in certain aspects of teaching	Embedding of new skills Freedom to learn from mistakes Exploration of relational aspects	Holding Ability to move between a more structured approach and a more collegial approach
Proficient/advanced	Increased confidence Greater insight	More reflective More exploratory Challenging of teaching and practice Broader themes	More collegial May be peer-based

## The Middle Circles—the Content of Supervision

The content and themes brought to supervision fall into four areas identified in the middle circles: teaching skills, theory/understanding, participants/the group and personal practice/process (see Fig. 1). The developmental phase of the supervisee plays a strong part in determining what they bring to supervision. This is particularly evident with newer teachers where there is a strong emphasis on developing understanding of the elements and intentions of the programme they are teaching. Supervisees often bring to supervision issues that have emotional charge, a sense of confusion and/or a lack of confidence. Because there is potential within supervision to develop a sense of creativity and playfulness, rather than tension, it is important to allow new perspectives to emerge, as shown in the first example in Table 3. This table outlines the middle circles giving some examples and illustrations from the supervisor interviews.

**Table 3:** Middle circle examples

Middle circles	Examples of what might be brought to supervision	Interview example
Teaching skills	The curriculum, leading practices, making CDs, timing, conveying teaching, handouts, resources, all the preliminaries before even beginning to run a course, feedback on teaching	The person I can think of was having tremendous difficulty not getting everything upside down and back to front. So it was to practice the order, the sequence of the guidance. And to do it in a place that felt quite safe rather than the anxiety of the class. (Sally 2012)
Theory/understanding	Theory from MBCT/MBSR, broader understandings of mindfulness or Buddhist understandings	They said, “What’s the intention behind doing body work again in MBCT?” And when you get questions of that order it’s a reminder that that is where this person is at and they need a real kind of immersion in the course and what all its different components are. But with some inquiry of what do you feel when you do the movement practice. (Lucy 2012)
Participants/the group	The relational aspects of teaching, the individuals in the class, the group process, and co-teachers, often aspects of the inquiry	I was supervising somebody on Friday and one of the participants was taking him off on a very intellectual stream and the question was how to come back from that, how to not just let it burble on but how to bring the focus back to the present moment. (Sally 2012)
Personal practice/process	Particular issues/struggles in relation to practice, reflections on practice, overwork, busyness, confidence issues	Sometimes I am helping to evolve practice- giving pointers, working with distraction or the inner critic or, if people haven’t been meditating for that long and are much newer to the whole thing, I have suggested particular approaches that might be helpful for one’s own practice and also supporting teaching. (Rachael 2012)

Material comes into the supervision space in various forms, including verbal reports, written reflections, teaching and inquiry of mindfulness practice. The use of DVD recordings of teaching practice within MBS seems to be growing. Although it can be challenging and exposing for supervisees to share their practice in this way, it holds many possibilities. “Many teachers lack confidence, so seeing themselves on DVD allows the supervisor to support what went well. Supporting people’s confidence that they didn’t make a dogs dinner out of it.” (Rachael)

Rachael discussed her own experience of showing her supervisor DVDs: “My first experience was very challenging because, you know, she was far more critical of it than I expected, and I didn’t know half the things I could have been doing better. It was extremely useful.”

One of the major advantages of viewing teaching practice with a DVD is that supervision becomes grounded in the actuality of what is happening in the teaching space. Without it, there is reliance on self-report from the supervisee, which is inevitably clouded by subjective perception. Body posture and gestures can come to light, such as the crossing of legs, fidgeting with papers, eye contact or lack of, grimaces and contractions, that all provide information for fascinating inquiries.

I can think of somebody I saw recently who had a great pile of notes with her when she was teaching that she kept looking at. She hadn’t told me she was looking at them. It turned out it hadn’t even occurred to her that it wasn’t a good idea. And I was saying you need to let go of your notes. And when she did it made a huge difference. (Emily)

There are limitations to the use of DVDs. Supervision also needs to give space to a deeper connection and inquiry into the supervisees’ and participants’ process which is not visible on a DVD.

Woods (2010) described two interlinked strands within mindfulness-based supervision, one of supervision of teaching and another of sustaining and deepening personal mindfulness practice. Although some teachers have a separate mentor/teacher to explore practice, many utilise supervision for personal practice development as well as teaching practice development. These decisions may be made based on the skills of the supervisor, the needs of the supervisee and personal preference. The important aspect is that both strands are addressed.

## The Inner Circle—the Supervision Space

### Holding the Supervision Space

This inner circle reflects the creation of a safe space where issues are brought, explored, integrated and then taken back to teaching, practice and life. It is a space that is ‘held’ and structured through clearly defined roles, timeframes and a written contract. The contract will involve the supervisor, supervisee and other relevant parties (e.g. the training organisation that the supervisor works for). The contract may include practical issues such as time and length of sessions, payment, recording and storage of notes and the review process. It also importantly clarifies confidentiality, expectations, accountability, roles and the intentions of the supervision. The processes which ‘hold’ and create a sense of safety are important because without them, the supervisee would not be enabled to move towards challenges or to be playful with experience.

The supervisors interviewed described their different preferences and styles around developing the agenda and session format. Some ask supervisees to e-mail an agenda in advance whilst others agree through discussion at the beginning of the session. There may be a need to negotiate what is on the agenda and prioritise. It is useful if the supervisee engages in a pre-supervision practice where they reflect on the issues in advance of the session. This short reflective practice may give the supervisee a felt sense of what is arising uppermost in relation to their teaching or practice. Although in general, supervisees bring the agenda items, there will be times that a supervisor does. This can occur, for example, when the supervisor wants to continue something from a previous session or to raise concerns about some aspect of practice.

There is generally a session format as well as an agreed, flexible structure to help encourage responsiveness to the moment. The sessions may include, for example, a period of practice (strengthening the container of mindfulness either in the session or beforehand); agenda setting; a review from the last session; space to inquire and reflect, acknowledging the learning, identifying what to take forward; and a summary of the session. These structures give scaffolding to the process of supervision but it is also important to maintain freshness by not getting stuck with one formula or with one supervisor. Regular reviews of the process at the end of each contracted period support freshness of approach. Keeping clear boundaries is also important, because although there may be an element of friendship in supervision, this is not its primary purpose. Finally, confidentiality needs to be very clear as supervisor and supervisee may have several colleagues and acquaintances in common.

### What Happens in the Supervision Space

#### Mutual Inquiry

A key vehicle for learning within MBSR/MBCT classes is the inquiry process (Crane 2009; McCown et al. 2010), and this is the same for supervision. “Ideally the whole supervision is a kind of mutual inquiry into the group and what happened, why and what went well” (Rachael). This mutual engagement in a process of exploring, reflecting and getting curious about all aspects of teaching was emphasised by several interviewees. “I strongly believe that we can’t do this exploration on our own. The inquiry needs nourishment. It’s a relational exploration” (Diane). The relational process supports a steadiness of practice and attention as well as a willingness to linger in a space of uncertainty and ‘not-knowing’ at times.

Inquiry is an interactive and relational way of verbalising curiosity about experience. In doing so, there is space for that curiosity to develop as we form and hear the words from ourself and others and feel the impact on our body. We are exploring experience through our ‘being’ mode of mind. A simple example from an interviewee was to bring a breathing space into the session in the midst of an exploration about teaching practice. This was followed by an inquiry along the following lines of: “What is going on with your body now? How are you working with this? How do you work with this in your teaching?” (Julia). The inquiry thus opens out in layers beginning with direct experience, then to dialogue and reflection and opening out to linking with teaching and, then, finally, back again to direct experience and so on (Crane 2009). There can be a tendency during MBS to move the inquiry too quickly to conclusion or to premature summing up. Several interviewees pointed towards the importance of lingering in the exploration: “So this real burrowing into something that caught their attention, and getting curious about it together …really draws me into curiosity. Inviting and questioning, being together in the not knowing” (Charlotte).

It can be easy for supervision to become filled with the content of what happened, but the primary intention is to move away from narrative and towards investigating and becoming curious. Even with newer teachers who want to learn the programme form, an inquiring approach is still employed using open-ended questions such as the following: “Why are we doing this? What is the intention? What is your feeling about it? What is happening in the classroom for you? How are you noticing your interactions with people? Where do you get most anxious? Where do you settle in to the present moment? What supports you?” (selection of examples from interviewees).

#### ‘Present Moment Focus’

What I like to know is what’s going on inside people while they are teaching and whether they have a very strong sense of the practice of mindfulness itself as the basis for the teaching. Instead of it being form orientated, it is more essence orientated. The essence of paying attention and being really present. (Charlotte)

Although inquiry in supervision is often focused on the exploration of past experience, there is an intention to keep bringing the exploration back to the immediacy of this moment since learning and integration so often emerge through this connection. Supervisors may incorporate ways to pause, connect and sense into direct experience in the midst of dialogue. The supervisor holds this delicate balance and chooses when to move the process in one direction or the other. As supervisees talk about some aspect of teaching, there is always the possibility of exploring the inner landscape of their experience as they recall and recount situations, moving from the attention on the story to what is underneath it. There may also be times when supervisors share the immediacy of their present moment experience as part of this exploration of inner landscapes. Both parties are actively involved in this inquiry and learning together. By practising this way of being present in supervision, supervisees learn how to be with another in the present moment during dialogue. Through this, they can develop awareness of their inner landscape and trust in the process of mindfulness. Consequently, the process of developing mindfulness whilst in a relational context becomes more natural and intuitive in the midst of teaching classes as described here: “You [the supervisor] are not trying to fix them [the supervisee]. You are not trying to draw out the narratives of their problem. You are being with them to keep helping them to look at what is actually happening in the present moment.” (Charlotte)

For some supervisees this is a difficult process because the automatic pull back to habitual styles is strong:

I find I might encourage them to what’s underneath, but somehow it just keeps flipping back. I will bring in a pause or a breathing space. I will specifically say, “Just pause now and drop into your experience and notice.” I have a supervisee in mind at the moment where I have an image of a rubber band which just keeps pinging back, but that’s the intention to keep redirecting our attention to underlying process. (Diane)

#### Integration and Linking

Ryan (2008) described an important quality of ‘looking’ within mindfulness supervision, in terms of how we wake up a creative space and reconnect with innate qualities that are often already there. The primary process in MBS is not the supervisor sharing the wisdom and experience that they have (although this sometimes happens), but rather the supervisor is enabling the supervisee to discover and connect to their intrinsic knowing. When inquiry and reflections on mindfulness and teaching are grounded in the present moment, there is a deeper connection with experience and a movement towards fresh seeing, which in turn may enable new learning to emerge. Within MBS, connecting to a felt sense in the body is an important part of exploring a new way of understanding a situation.

Table 4 shows an example of an inquiry within a peer supervision that illustrates particularly what was happening within the supervision space, moving from direct experience through to a wider context of understanding and, then, returning back to teaching.

**Table 4:** Example from a peer group supervision session

Link with the framework	Experience in the supervision
Outer circle	Mindfulness container held by the group. There was a short practice to start the session to orientate to a sense of being. Linking to mindfulness, embodiment and compassion continues throughout the process
Middle circle—the content brought to supervision	A participant had missed a couple of sessions and did not seem very engaged. The supervisee noticed they were not very actively following this up
Inner circle (present moment)	In the supervision, a noticing of a feeling arises, a contraction in the body around the abdomen, a feeling of harshness and irritation, giving up on the participant, almost feeling it would be easier if they left
Inner circle (mutual inquiry with peers)	Contemplating the question as to what might be happening for this participant underneath what is presented, a sense of their vulnerability
Inner circle (present moment)	Supervisee noticing body sensations softening and opening, more space in the body and mind
Inner circle (seeing from a fresh perspective, making links)	Seeing dissatisfaction/suffering for the participant and self. Seeing and feeling the lack of compassion and the possibility of opening in a different way
Inner circle (integration and action)	Making a decision to phone the participant, coming from an open compassionate stance. This decision coming from a bodily felt sense, feeling congruent with the exploration
Taking the learning out in to teaching (continuing to integrate learning)	The supervisee’s ability to soften towards this participant continued and they did complete the course

The first author’s own experience

Interviewees spoke of supervisees expressing an appreciation of MBS and the way it informed positive changes to teaching practice. For a supervisee who is less experienced, the link to teaching is often more tangible, whilst for experienced teachers, there are increasing levels of subtlety and nuance. Supervisees often report how they have experimented with teaching differently following supervision. They describe feeling supported; more confident in their teaching and their own practice; and feeling less stressed, pressured or burnt-out. Others report how supervision reawakens a sense of freshness and aliveness to their personal and teaching practice. Supervisors see and hear these changes on DVD in the way people sit, the language they use and the clarity of their teaching.

Practising mindfulness and teaching mindfulness is deeply challenging work because MBI teachers are supporting participants to turn towards and work with often very difficult mental and physical states. It would not be possible to sustain either without the support and inspiration that we draw from others who are engaged in this way of exploring human experience. Supervision is a purposeful and focused way of offering and receiving this support and inspiration.

## Discussion

This paper presents our conceptual ideas about a framework for MBS describing the structure, content and process of supervision based on current practice. The exploration presented in this paper was limited in scope to articulating the process of MBS as it has emerged within the field during this period of rapid change and expansion. In presenting our perspectives on MBS, we hope to offer some clarity about the nature, role and purpose of supervision for mindfulness-based teachers as it is perceived by practitioners in the UK and to stimulate dialogue and inquiry on this important issue. At a recent MBS training day at a UK university, the framework was presented and explored with supervisors. The initial feedback was positive, showing an interest in using the framework. One participant on the training day commented, “I think your diagram will be very helpful and look forward to using it with a series of supervisions I am about to do.”

This was not a formal research project so the lack of methodological rigour limits the validity and generalisability of the framework. It would be particularly useful if further qualitative investigation is conducted around MBS. Particular areas for investigation maybe comparing supervision models that are developing in the context of mindfulness-based teaching with models used in other related areas such as cognitive behavioural therapy. It would also be useful to compare what is presented here with models that have emerged in other countries (as this is primarily based on the UK experience).

As the interest in mindfulness-based approaches expands, the field faces some challenging developmental issues related to responding to the demand whilst also attending to the integrity of mindfulness-based interventions as envisaged by their developers. Jon Kabat-Zinn (2011) stated, “....MBSR is at its healthiest and best when the responsibility to ensure its integrity, quality and standards of practice is being carried by each MBSR instructor him or herself” (p. 295). In our view, supervision is a key way to nurture the development of individual teachers so that they can resource themselves to teach with integrity. This will inevitably support the development of the field.

There are some challenges in ensuring that there are enough supervisors able to take on this role. The role of the mindfulness-based supervisor is a complex and challenging one, requiring significant experience of mindfulness-based teaching and training in supervision. It is important that supervisors are adequately trained, skilled and supported in this role.

A further challenge is that access to supervision may be restricted by a teacher’s ability to fund it. Significantly, in a recent UK survey of MBCT teachers (Crane and Kuyken 2012), a chasm was identified between standards set for training and supervision and the reality of 66 % reporting not having ongoing support in the form of continuing education or supervision (probably due to time and financial constraints). This highlights the importance of building the cost of supervision into the running of classes and the setting up of services wherever possible.

Many teachers come to teach MBSR/MBCT with a heartfelt intention to engage in a way of life and livelihood that has meaning and supports their own well-being as well as that of others. Supervision may be one aspect of nurturing this intention.

In summary, supervision for MBSR/MBCT teachers is a place to foster curiosity; engage in dialogue about mindfulness teaching experiences in the past and present moment; promote integrity; experience warmth, compassion and mentorship; be supported, challenged and human; and to make sense and meaning in a way that benefits others. In short, supervision is a place for embodied mutual inquiry that cultivates mindfulness and the benefits it can bring.
